# A neural network analysis of the effect of high and low frailty index indicators on predicting elective surgery discharge destinations

**DOI:** 10.1371/journal.pone.0284206

**Published:** 2023-04-07

**Authors:** Steven Walczak, Vic Velanovich

**Affiliations:** 1 School of Information & Florida Center for Cybersecurity, University of South Florida, Tampa, Florida, United States of America; 2 Department of Surgery, Morsani College of Medicine, University of South Florida, Tampa, Florida, United States of America; Yale School of Medicine: Yale University School of Medicine, UNITED STATES

## Abstract

**Background:**

Frailty is frequently used by clinicians to help determine surgical outcomes. The frailty index, which represents the frequency of frailty indicators present in an individual, is one method for evaluating patient frailty to predict surgical outcomes. However, the frailty index treats all indicators of frailty that are used in the index as equivalent. Our hypothesis is that frailty indicators may be divided into groups of high and low-impact indicators and this separation will improve surgical discharge outcome prediction accuracy.

**Data and methods:**

Population data for inpatient elective operations was collected from the 2018 American College of Surgeons National Surgical Quality Improvement Program Participant Use Files. Artificial neural network (ANN) models trained using backpropagation are used to evaluate the relative accuracy for predicting surgical outcome of discharge destination using a traditional modified frailty index (mFI) or a new joint mFI that separates high-impact and low-impact indicators into distinct groups as input variables. Predictions are made across nine possible discharge destinations. A leave-one-out method is used to indicate the relative contribution of high and low-impact variables.

**Results:**

Except for the surgical specialty of cardiac surgery, the ANN model using distinct high and low-impact mFI indexes uniformly outperformed the ANN models using a single traditional mFI. Prediction accuracy improved from 3.4% to 28.1%. The leave-one-out experiment shows that except for the case of otolaryngology operations, the high-impact index indicators provided more support when determining surgical discharge destination outcomes.

**Conclusion:**

Frailty indicators are not uniformly similar and should be treated differently in clinical outcome prediction systems.

## Introduction

Prediction of surgical discharge outcomes prior to an operation is beneficial to clinicians, post-operative care providers, patients, and their families [1]. Therefore, it is important to only use independent variables in the prediction model that are available prior to surgery to enable effective post-operative care planning, improved recovery, and stress reduction for patients.

The frailty index (FI) has been shown to be an effective indicator of surgical patient outcomes [2, 3], especially with elderly patients [4], or patients whose health is otherwise compromised affecting their ability to cope with stressors [5]. The accumulating deficits FI model of Rockwood and Mitnitski [6, 7], which represents frailty as a single value composed of the sum of all deficits present in a patient divided by the total number of deficits measured, initially recommended having 40 or more deficit variables. Prior research however has shown that a much smaller collection of variables is able to produce reliable FI and these smaller variable models are referred to as modified FI (mFI) [8–10].

The frailty indicator variables used in our research to construct the mFI are:

- ascites,
- dialysis within 2 weeks,
- functional status,
- history of chronic obstructive pulmonary disease within 1 month,
- history of congestive heart failure within 1 month,
- renal failure within 24 hours,
- ventilator dependent,
- current smoker within 1 year,
- diabetes,
- disseminated cancer,
- dyspnea,
- high blood pressure or on hypertensive medications, and
- steroid use within 1 month.

These variables are taken from previous studies [8, 9, 11], which have mapped the original FI values [6, 7] onto an existing national database of performed operations. Furthermore, [8, 9, 11] have demonstrated the efficacy of using the listed indicators in calculating a mFI for surgical outcome prediction. A glossary of medical terms is provided in the S1 Appendix.

The different indicators used in the mFI have been shown to have varying influences on the true degree of frailty [12, 13], but mFI is traditionally treated as a single value with all indicator variables contributing equally. Prior research demonstrates that the presence of specific preoperative comorbidities (all of which would be counted equally in a mFI) are associated with increased occurrences of specific complications and the effect of the presence of additional comorbidities increased the frequency of the occurrences of this complication more than what would be predictive based on merely adding the risks together, i.e., the greater-than-additive phenomenon [14].

Our research motivation is to see if among the multiple indicators used to create mFI, some pose greater risks of adverse outcomes, which limits the accuracy of current models using mFI to predict outcomes where all indicators are treated equally. We define those items within the mFI that have greater influence on predictability of negative postoperative outcomes as high-impact indicators, while those that have a lower influence on predictability as low-impact indicators. The high-impact indicators are the first 7 indicators listed above from ascites to ventilator dependent, with the remaining 6 (current smoker to steroid use) being defined as the low-impact indicators. The differentiation of indicators into high-impact and low-impact is determined by the individual indicator’s influence on 30-day postoperative mortality as shown by Madsen et al. [15]. The consequent research hypothesis is:

H_0_: Surgical outcome prediction models that treat high-impact frailty indicators differently from low-impact frailty indicators will increase outcome prediction accuracy.

Evaluating surgical outcomes may be done using a variety of measures including length of stay and mortality, however a more recent approach that overcomes issues with length of stay such as lack of social and rehabilitative services has been proposed: discharge destination [16]. Prior research has noted that using discharge destinations is an important metric for evaluating quality of care [17]. Therefore, the patient’s discharge destination is used as the dependent prediction variable and should be considered as the outcome for H_0_.

The objective of our research is to examine if high-impact indicators affecting frailty and other low-impact indicators used in a mFI, affect prediction of surgical outcomes differently as measured using hospital discharge destinations. Because of artificial neural networks (ANN) effectiveness in evaluating new heuristics, this research question will be answered by using ANN models to evaluate the new heuristic of treating high-impact and low-impact indicators differently when used for predicting surgical discharge destination outcomes [18]. The ANN evaluation is performed by directly comparing changes in destination prediction accuracy and is enhanced by using the leave-one-out training strategy to identify high-impact and low-impact indicator importance [19, 20].

## Materials and methods

This study used anonymized historic data from a large national database freely available to member hospitals participating in the American College of Surgeons National Surgical Quality Improvement Program (NSQIP). Due to the use of retrospective anonymized data this study was given a waiver for need for consent from the USF Office of Research Integrity and Compliance.

All data is acquired from the American College of Surgeons National Surgical Quality Improvement Program (NSQIP) for the 2018 Participant Use Data (PUF) files, which has 1020511 available records reported from 722 hospitals across 49 states in the United States and 11 other countries [21]. The data is constrained to only use elective inpatient operations to provide a more homogenous patient group as trauma and emergency surgery patients have categorically different preoperative physiological insults and often have ongoing disabilities following surgery which affect discharge destination [22]. Using only inpatient elective operations reduces the total number of NSQIP surgical records available to 390185. The only exclusion criterion is those records missing values for the corresponding independent variables for the ANN model.

ANN models are used to evaluate the effect of splitting the mFI factors into high-impact and low-impact factors [18]. Each model has one of: original mFI, high-impact and low-impact mFIs jointly, high-impact only mFI, or low-impact only mFI; where the mFIs are calculated as described in the Introduction. Additionally, each ANN model also incorporates independent variables for age, sex, work relative value unit (wRVU), and ASA (American Society of Anesthesiologists) class [23]. These additions to the mFI as independent variables are due to age and sex commonly being used in frailty models [12, 13], wRVU which represents the length of an operation and is used as a measure of the degree of stressors experienced, and ASA class because of its use in the NSQIP surgical risk calculator [24].

Data is collected from the NSQIP data files for 9 different surgical specialties: cardiac, general, gynecology, neurosurgery, orthopedics, otolaryngology (ENT), thoracic, urology and vascular as well as combining them all together in a composite set of all operations to create 10 datasets. Each dataset is first divided into two unique sets: one for training and one for testing each model, with the discharge destinations for each surgical specialty divided evenly between these sets and records selected randomly within destinations. Patient demographics for the composite training and test sets, as well as the complete inpatient elective surgery population, are shown in Table 1. The differences in demographics between the training and test sets are small and none are statistically significant, based on Z-test results.

**Table 1:** Demographics for composite training and test sets.

	Training set	Test set	Population
% male	50%	49.9%	43.8%
work RVU (η ± σ)*	20.7 ± 10.4	21.16 ± 10.8	20.7 ± 8.6
age (η ± σ)	66 ± 17.0	68 ± 16.4	63 ± 14.6
ASA class (η ± σ)	2 ± 0.81	3 ± 0.88	3 ± 0.64
mFI (η ± σ)	0.08 ± 0.09	0.08 ± 0.10	0.08 ± 0.08
mFI high-impact only (η ± σ)	0 ± 0.07	0 ± 0.08	0 ± 0.04
mFI low-impact only (η ± σ)	0.17 ± 0.16	0.17 ± 0.17	0.17 ± 0.15
% discharge to home	14.5%	15%	91.1%
% discharge to home with facility	14.3%	14.6%	0.50%
% discharge to multi-level facility	2.0%	1.5%	0.02%
% discharge to unskilled facility	8.0%	6.8%	0.1%
% discharge to rehab	14.5%	15%	2.9%
% discharge to skilled facility	14.5%	15%	4.8%
% discharge to acute care	14.2%	14.5%	0.3%
% discharge to hospice	5.2%	4.8%	0.05%
% mortality	12.7%	12.9%	0.3%

* η (median), σ (standard deviation)

The training and test sets for the ANNs are then partially balanced to limit overlearning of a smaller number of output classes [25]. Partial balancing is performed by calculating the median of the number of real-world destinations for each type of surgery, with specific destination quantities above the median limited to the median number, and destination quantities below the median utilizing all available cases, for each of the 9 surgical specialties. The selected records for destinations limited by the median value for all destinations are chosen randomly to eliminate any selection bias.

Single hidden layer, two hidden layer, and three hidden layer architectures are developed, with the number of hidden nodes in each hidden layer ranging from $\frac{1}{3}n$ to 4*n*, where *n* is the number of nodes in the preceding layer. Layer node augmentation continues until two successive increments result in reduced performance on the test set data [26]. Training, using backpropagation supervised learning, occurs over a minimum of 50000 epochs up to 150000 epochs in steps of 10000 until the root mean square error (RMSE) of the training set falls below 0.05 or the RMSE is consistent over 10000 training iterations. Other best practices for ANN design as specified in the research literature [26, 27] are followed to ensure high quality and reproducible ANN models.

Neural networks are developed separately for each of the nine surgical specialties and the composite set of all surgeries regardless of specialty. Each specialty/composite has both a mFI (as a single value) and separated high-impact and low-impact mFI (2 values, with both used together as independent variables) ANN model. A leave-one-out analysis is performed with the variables left out being the high-impact mFI and the low-impact mFI from the dual high and low mFI ANN model to evaluate the relative contribution of each mFI on overall prediction accuracy.

### Statistical methods

The goal of determining if the mFI may be split into two distinct categories is evaluated by comparing the respective models for prediction accuracy, measured against the real-world hospital discharge recorded in the NSQIP data file. An increase in prediction accuracy is considered confirmatory for the exploratory research hypothesis. Further evaluation is performed to determine if noted differences in destination prediction accuracy may be considered statistically significant by using a standard one-tailed Z-test for differences, with significance reported as *p*-values. A 95% confidence interval is used with significance denoted by *p*-values less than 0.05 and high significance denoted by *p*-values less than 0.01 [28].

## Results

The best performing ANN architectures for each specialty and corresponding mFI configuration, are given in the S2 Appendix. Results of the 20 ANN discharge destination prediction models, utilizing either a single mFI or split high-impact and low-impact mFIs is given in Table 2, with percent improvement of the split high-impact and low-impact mFIs over the traditional model calculated as:

$$\frac{2\;impact\;class\;mFI\;accuracy}{single\;mFI\;accuracy}-1$$ (1)

**Table 2:** ANN destination discharge prediction accuracy.

Surgical Specialty	mFI (single value)	high-impact mFI & low-impact mFI combined	% Improvement
Cardiac	30.77%	26.92%	-12.5%
ENT	28.81%	32.20%	11.8%
General	34.83%	44.03%	26.4%
Gynecology	33.04%	41.74%	26.3%
Neurosurgery	39.93%	51.16%	28.1%
Orthopedics	35.00%	36.18%	3.4%
Thoracic	33.60%	38.40%	14.3%
Urology	47.03%	54.34%	15.5%
Vascular	26.56%	32.19%	21.2%
Composite (all specialties)	31.99%	36.80%	15.0%

### Leave-one-out experiments

**Fig 1:**
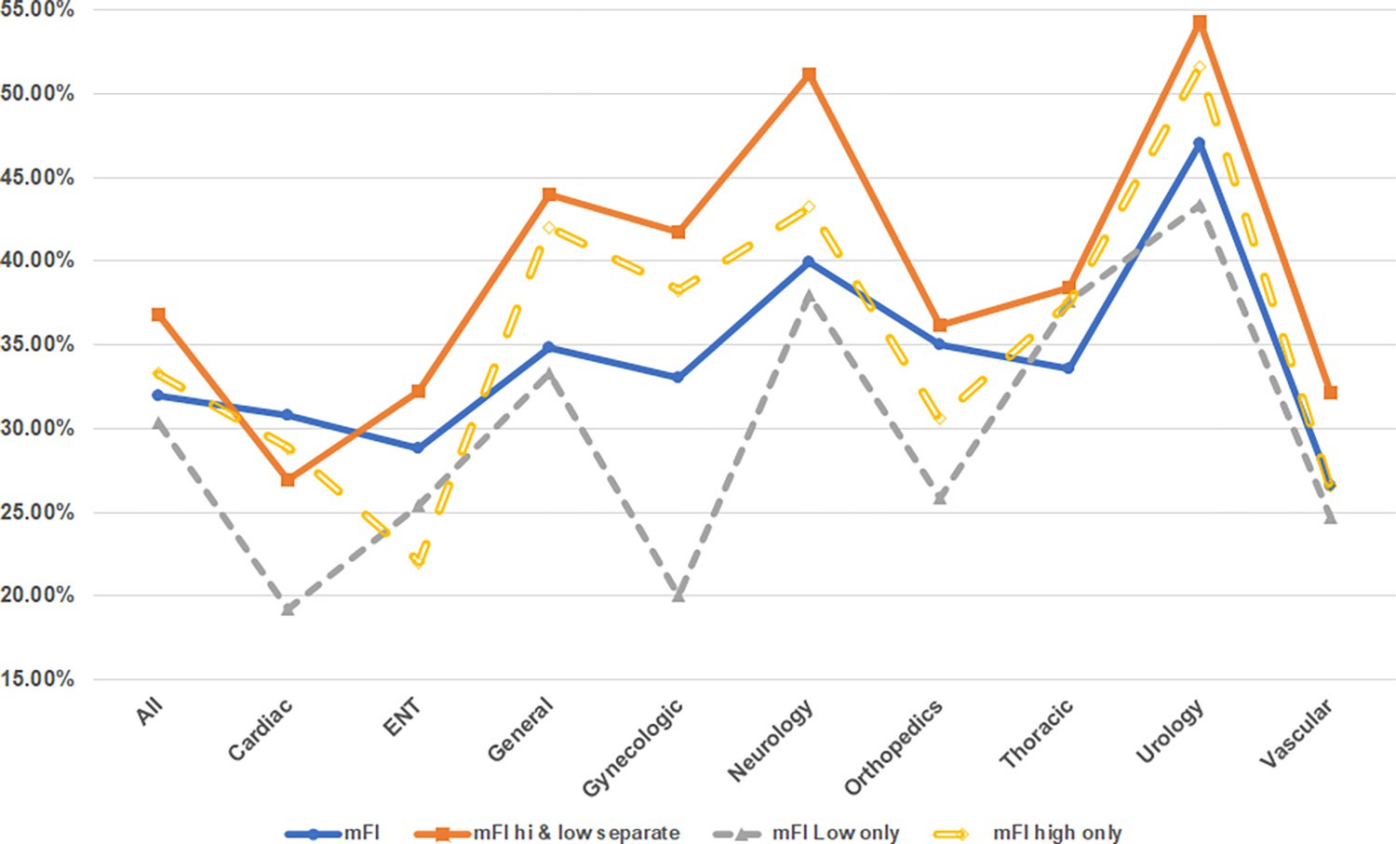
Leave-one-out comparison of ANN prediction accuracy against original models.

The relative prediction accuracy of the individual low-impact indicator mFI and the individual high-impact indicator mFI compared to the original single mFI (using all indicators) and the combined high-impact and low-impact mFI ANNs is shown in Fig 1.

### Cross model statistical significance summary

Four distinct backpropagation trained ANN models have been reported for each of the 9 operation specializations as well as for the combination of all surgery types specified. Table 3 displays the statistical significance, or lack thereof, for comparing the relative prediction performance improvements between the model that uses a single mFI with all indicators treated equally and the model that splits the indicators into separate high-impact and low-impact that enables the ANN to assign different weights for each mFI variable being transmitted to the first hidden layer. Table 3 also shows the significance for differences between the models that utilize only the high-impact indicator mFI or the low-impact indicator mFI. Bold red values in the table indicate any significance in the opposite direction.

**Table 3:** Statistical significance of prediction accuracy differences, reported as *p*-values.

Surgical Specialty	Split High-impact and low-impact indicator mFI models improvement over traditional mFI models	High-impact mFI only model improvement over low-impact mFI only model
Cardiac	**0.3324**	0.1241
ENT	0.3445	**0.3325**
General	0.0037*	0.0053*
Gynecology	0.0856	0.0009*
Neurosurgery	0.0026*	0.0925
Orthopedics	0.3743	0.0861
Thoracic	0.2143	0.5
Urology	0.0626	0.042**
Vascular	0.0588	0.2935
Composite (all specialties)	0.0008*	< .0001*

* highly significant improvement, *p* < 0.01

** significant improvement, *p* < 0.05

### Corollary experiment

How comfortable will surgeons be working with two frailty index values? Utilizing the outcome of the ANN destination prediction research shown in Table 2, an attempt to combine the significance of treating high-impact and low-impact factors differently into a single mFI is evaluated through this corollary experiment. While a traditional accumulating deficits mFI sums all deficits and divides by the number of possible deficits, incorporating the differences between high-impact and low-impact indicators may be done by multiplying the high-impact indicators, which as shown in Fig 1 have greater impact on discharge destination predictions for all models except the ENT surgical specialty, by a specified constant *β*. Values of 1.5, 1.667, 1.75, and 2.0 are used as possible values for *β* in the formula:

$$\hat{mFI}=\frac{\sum (\beta *high-impact\;indicators)+\sum (low-impact\;indicators)}{total\;number\;of\;factors}$$ (2)

The *β* multiplier effectively increases the influence of the high-impact indicators in the new $\hat{mFI}$ index term to simulate the differentiated weighting achieved using separated high and low indicator values in the ANN model, each of which has its own set of weighted connections to the first hidden layer of the ANN.

**Fig 2:**
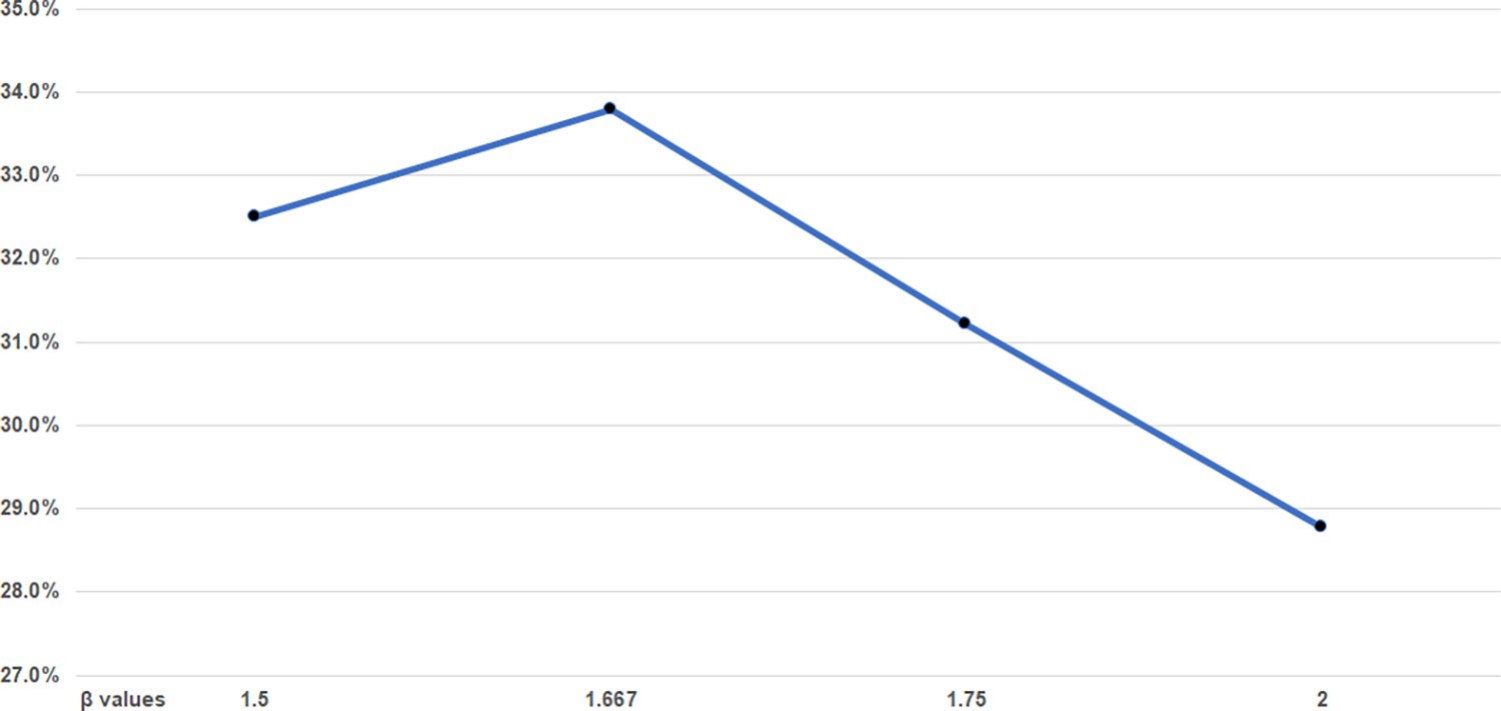
Destination prediction accuracy for different values of β in $\hat{mFI}$.

ANN prediction accuracy for the composite set of all operations using the $\hat{mFI}$ (also known as mFI-hat) with different *β* values are shown in Fig 2. The new $\hat{mFI}$ may produce an index of greater than 1, but for all elective operations used in this study, the $\hat{mFI}$ values ranged from 0 to 0.795. Since the *β* value of 1.667 produced the best prediction accuracy over the composite set of all surgeries, this value is uniformly used for all other operations.

**Fig 3:**
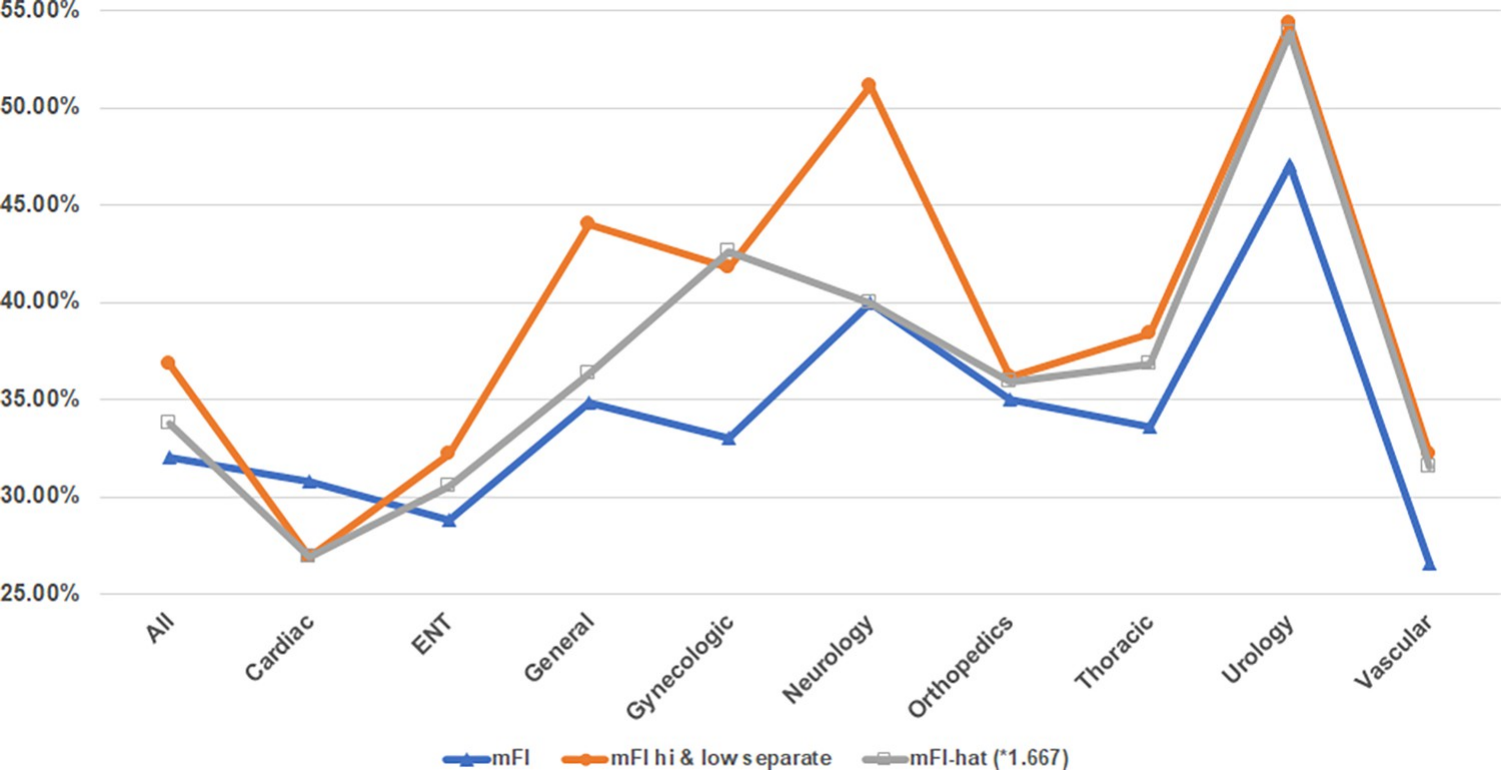
Comparison of ANN model’s destination prediction accuracy utilizing traditional mFI, new 2 value mFI, and $\hat{mFI}$.

When the $\hat{mFI}$ is used as the variable in place of the original mFI or the 2 high-impact and low-impact mFI variables model, the new prediction accuracies are shown in Fig 3. As may be seen in Fig 3, the new $\hat{mFI}$ ANN produces prediction accuracies between the original mFI and the new two value high-impact indicators and low-impact indicators mFIs, including being equal or nearly equal to one or the other for cardiac, neurology, orthopedics, and urology.

## Discussion

The reported research has demonstrated that for most types of operation, the splitting of an mFI into two mFI (one for high-impact indicators and one for low-impact indicators) used in an ANN model where different weights are applied to each of the separate mFI indicator types produces improved accuracy for destination discharge outcome predictions. This directly supports our H_0_ research hypothesis for eight out of the nine surgical specialties studied and for the composite set of all surgeries. The cardiac surgical specialty results contradict H_0_. If H_0_ is rewritten as 10 new hypotheses H_1_ to H_10_ including the surgical specialty, such as: Surgical outcome prediction models that treat high-impact frailty indicators differently from low-impact frailty indicators will increase outcome prediction accuracy for general surgery, then 9 of the 10 hypotheses are confirmed and only 1 is rejected.

The increased prediction accuracy is statistically significant at *p* < 0.01 for two of the nine surgical specialties and for the composite set of all operations. The leave-one-out experiment determined that the high-impact indicators mFI outperformed the low-impact indicator for eight of the nine surgical specialties and the combined set of all operations.

There are two important clinical impacts of these findings. First, the presence of high-impact items can provide targets for prehabilitation. Prehabilitation refers to efforts to improve aspects of the patient’s physical and cognitive condition prior to surgery [29], many of which contribute to frailty. While efforts directed toward the lower impact items should not necessarily be ignored, greater reduction in adverse outcomes should be expected by correcting the high-impact indicators. Second, by identifying patients at risk of discharge to higher level of care destinations prior to surgery, hospital resources can more effectively be employed, reducing administrative and clinical burden without impacting outcome. Clinical decision support systems utilizing either the two index ANN or a scaled $\hat{mFI}$ value can be integrated into the hospital’s electronic medical record’s decision support system to help clinicians and the appropriate hospital staff identify at risk patients [30].

The indicator items deemed high-risk based on their influence on postoperative mortality [15] (dependent functional status, ventilator dependent, history of chronic obstructive pulmonary disease, ascites, history of congestive heart failure within 1 month, renal failure within 24 hours, and dialysis within 2 weeks), all have a major impact on a patient’s physiologic reserve and their ability to withstand a physiological insult. Surgery, although aimed at improving a patient’s health upon recovery, is a short-term physiological insult of varying degrees. Although not inconsequential, the chosen 6 lower impact variables (diabetes, current smoker within 1 year, dyspnea, high blood pressure or on hypertensive medications, disseminated cancer, and steroid use within 1 month) do not have the same degree of physiological impact on the patient, as shown by their decreased prediction accuracy when compared to the high-impact indicators (Fig 3).

Performance improvement of the split high impact and low impact frailty indexes over the original single mFI for all surgical specialties excluding cardiac ranges from 3.4% to 28.1%, with an average prediction accuracy improvement of 18.3% across all surgical specialties including cardiac (19.1% improvement excluding cardiac surgeries). The performance improvement of the split high-impact and low-impact mFI values may also be interpreted as additional patients whose destination discharge is predicted accurately, thus improving post-operative clinical support for the patient. For example, the composite operations improvement of the split high-impact and low-impact mFI variables ANN yielded 125 additional correct destination predictions over the single mFI ANN model for the 1935 test cases, a 6.46% improvement. The cardiac specialty is the only one that did not show any improvement, but only results in 2 test set patients’ discharge destinations being misclassified by the new split impact factor ANN model (due to a smaller number of cardiac surgeries performed).

A major benefit of using ANN discharge destination prediction models is that the models, due to their nonparametric and nonlinear nature, produce individualized predictions for every surgical patient that accounts for minor adjustments in the presence of the various frailty indicators and other demographic values. ANNs facilitate generalized use of resulting models [31]. The use of a large international database from 722 hospitals to create the ANN training and test sets indicates that the results of the current mFI ANN models should be generalizable to most hospitals across the United States and within the country regions where NSQIP data originated.

### Mortality as an outcome

Perhaps the most important outcome for patient decision making on elective surgeries is mortality. All types of operations had cases of patients who died prior to discharge from the hospital. The composite set of all operations using the two distinct classes of frailty factors predicted 78% of all mortality results correctly.

### Leave-one-out insights

The leave-one-out experiment (Fig 1) shows the high-impact indicators only mFI has a more significant contribution to the prediction of discharge destinations for all but the ENT operations because its prediction performance accuracy is greater than the low-impact indicators only mFI ANN models. The ENT result may indicate that generally ENT operations produce smaller stress on the patient’s recuperative system. However, because the combined indicators ANN (both high and low-impact indicators separated and used) outperforms the leave-one-out models, this implies that both the high and low indicators are still needed for a robust discharge destination prediction model. This raises the question if the $\hat{mFI}$ index is used, then the β values used may be different for each surgical specialty.

### $\hat{mFI}$ application

The development of the heuristic $\hat{mFI}$ value that approximates the split high-impact and low-impact factors model into a single index serves as additional support for H_0_. As shown in Fig 3, the new $\hat{mFI}$ ANN model outperforms the traditional mFI for all types of operations, except cardiac, and even outperforms all other models for the case of gynecologic operations. Therefore, if surgical teams desire to still use a single mFI value for decision making, the new $\hat{mFI}$ should be used to emphasize the high-impact indicators that improve prediction accuracies of discharge destination outcomes. Additionally, this heuristic value may be easily incorporated into existing frailty models currently in use at hospitals simply by replacing the current mFI with the new $\hat{mFI}$.

### Destination coverage

**Fig 4:**
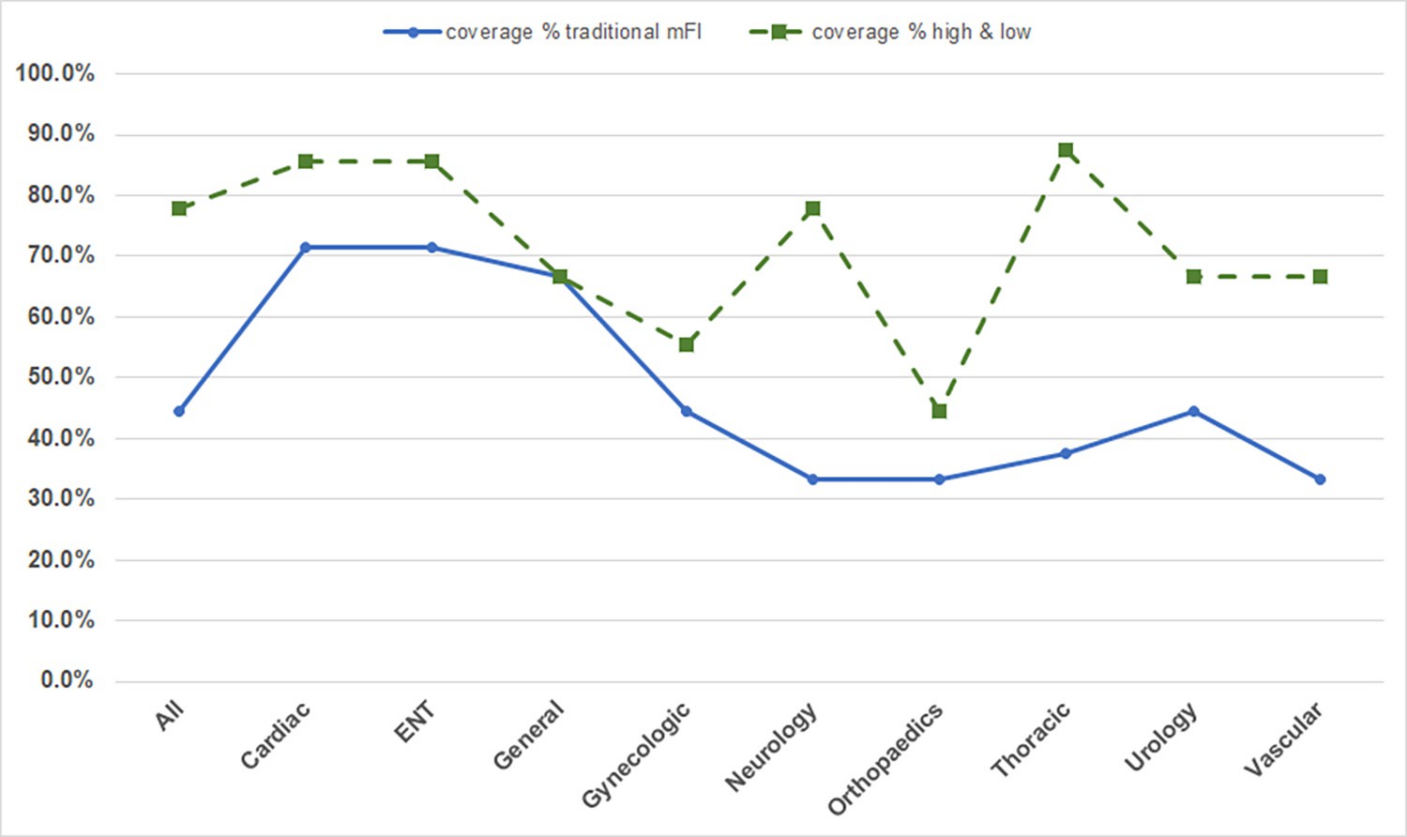
Percent of discharge destinations accurately predicted by the ANN mFI models.

The ability of the ANN models to predict destinations other than home is important as mentioned previously to provide knowledge for post-surgery care. It is important to understand that the various surgical specialties data from the NSQIP PUF data files do not always have at least two discharges to all 9 destinations, to provide an example for both the training and test data sets. The range is from 7 to 9 destinations, with the cardiac and ENT specialties each discharging to 7 destinations and the thoracic specialty discharging to 8 destinations, with all other surgical specialties discharging to the full set of 9 destinations. The ANN models all predicted multiple destinations and the split high-impact and low-impact model performed better than the original mFI model for all cases except general surgery where it performed the same. The distribution of discharge destinations, since not all specializations discharge to all destinations, covered by both models is illustrated in Fig 4. The split high and low indicator mFI ANN model is able to accurately predict greater than 50 percent of the possible discharge destinations for all surgical cases and surgical specialty cases except for orthopedic operations. Whereas the traditional single mFI ANN model only had coverage of 50 percent or greater of possible discharge locations for three of the 10 possible surgical case sets.

The multi-level care facility and unskilled care facility destinations are the two destinations that are most frequently missed by the split high-impact and low-impact mFI ANN model. These 2 destinations are among the three most infrequent discharge destinations following elective surgery as noted in Table 1. The hospice destination, the other least frequently discharged to location has more ANN models that predict this destination but is the next most frequently missed location by the ANN models. This correlation between the prevalence of discharge destinations and corresponding prediction accuracy supports the research technique of utilizing balanced data sets for training ANN clinical prediction models and indicates that future clinical ANN research models should all be developed using balanced training sets (or as near as possible given data limitations).

### Limitations

The first limitation is that the ANN prediction models are designed using inpatient elective surgery patient data only. Using only inpatient elective surgery limits the quantity of available data samples for training. Can similar results occur for emergency operations, or outpatient procedures?

A limitation on the development of the new $\hat{mFI}$ is that only a small number of multipliers (4) are tried and the peak performing value, with declines on either side, was selected. It is possible that another value between 1.0 and 2.0 may further improve the performance of the $\hat{mFI}$ and future research should seek to determine the optimal multiplier for high-impact frailty indicators.

### Future research

Further investigation is needed to determine why the split high-impact and low-impact mFI prediction models produce predictions for a larger percentage of discharge destinations than the traditional single mFI, except for the case of general surgery that has identical coverage of destinations by each of the ANN models. The question of what are the factors involving general elective surgery that led to this identical destination prediction coverage merits further research.

A question that surfaces is why the original mFI performed better at predicting cardiac specialty surgery discharge destinations than the split high-impact and low-impact mFI ANN model? This might be a byproduct of the smaller number of discharge destinations for elective cardiac surgery patients, which as reported is only 7. However, one of the discharge destinations only had two samples, meaning that only a single case is used for training and only one case remained in the test set, increasing the impact of an inaccurate prediction for this single case. If either of these cases was missing, then this would reduce the cardiac surgery specialty discharge destinations to 6, the lowest of all nine surgical specialties examined in this research. Cardiac operations tend to be of a fairly uniform anatomic insult. For example, as most cardiac operations in 2018 were done through a median sternotomy, mostly requiring intraoperative cardiopulmonary bypass, with manipulation of only the heart. Other specialties, for example, general surgery, have a more wide-ranging level of anatomic tissue insult, such that the impact of frailty may be more pronounced. There may be additional factors for how frailty affects cardiac surgery patients, and discovering a more robust set of frailty indicators for cardiac surgery patients is a topic for future research.

The overall prediction accuracy of the reported ANN models can be further improved by the addition of other reliable variables that indicate a patient’s ability to recover from the stressors of surgery [11] and through increasing the available data for training (and testing) the ANN models and is a topic for future research. Additional data will enable a larger number of real-world examples to increase the uniformity of presence of different destination outcomes, especially the ones with samples less than the current median enabling more robust training. Additional future research is needed to examine the incorporation of additional variables into ANN surgery outcome prediction models.

With respect to the inpatient elective surgeries used in our research, can similar results occur for other types of surgical procedures? Will different models be needed for traumatic versus medical emergency patients? Do patients undergoing outpatient procedures go to multiple different destinations with varying levels of postoperative care? These questions and the development of ANN models to predict discharge destinations for applicable operation types is a goal of future research.

The multiplier selected for the composite collection of all surgical specialties was the one used for all of the individual surgical specialties. It is likely as shown in Fig 1 and Table 2, that since the high and low frailty indicators have a different impact on each operation for each surgical specialty, that instead of using a single multiplier, the $\hat{mFI}$ might be further improved for predictions for a specific surgical specialty through specialization that would use a distinct β multiplier determined for each surgical specialty.

## Conclusion

While frailty has been shown to be a reliable indicator of surgical outcomes in prior research, the equal treatment of frailty indicators implies that every health deficit is identical regarding the ability for a patient to recovery from surgery. Our research demonstrates using ANN models, that frailty factors need to be divided into two groups: high-impact indicators and low-impact indicators. This knowledge discovery in medicine will facilitate improvement of clinical informatics systems for post-surgical prognostic planning and care which in turn will improve the health outcomes of elective surgery patients.

## Supporting information

S1 Appendix (DOCX)

S2 Appendix (DOCX)

S1 Data (CSV)
