# Supplementary material for: A neural network analysis of the effect of high and low frailty index indicators on predicting elective surgery discharge destinations
Source: PLoS One. 2023 Apr 7;18(4):e0284206. doi: 10.1371/journal.pone.0284206 (PMC10081744; doi:10.1371/journal.pone.0284206)
Supplement: S1 Appendix — (DOCX) [file pone.0284206.s001.docx]

**S1 Appendix**

**Glossary**

ASA class – the American Society of Anesthesiologists numeric value from 1 to 5 that corresponds to a subjective evaluation of a patient’s overall health status.

Ascites – fluid buildup in the abdomen, often caused by liver problems.

Dialysis – the exchange of blood in a patient to treat kidney failure.

Functional status – whether the patient is able to live on their own without assistance (independent), with some minimal amount of external assistance (partially dependent), or with significant external assistance (dependent).

Cancer (disseminated) – any cancer that has migrated to a second site.

Chronic obstructive pulmonary disease (COPD) – a progressive obstruction of the airways that makes breathing difficult.

Congestive heart failure – also known as heart failure is when the heart is unable to pump sufficient blood.

Diabetes – type 1 diabetes is the complete lack of insulin production by the pancreas which is a control for ketoacidosis, type 2 is partial or incomplete insulin production by the pancreas.

Dyspnea – shortness of breath often related to heart or lung disease.

Hypertensive medication – medication used to control hypertension also known as high blood pressure.

Ketoacidosis – acid in the blood produced by the liver when breaking down fats.

Renal failure – the kidneys are no longer able to filter waste from the bloodstream.

Steroid – a pharmaceutical that is used to control swelling and inflammation.

Ventilator – mechanical devices that move air into and out of the lungs.

Work relative value unit (wRVU) – is a value used by insurance companies for calculating physician compensation and represents a pre-defined amount of time that each operation is allowed for billing purposes.
