# Supplementary material for: A neural network analysis of the effect of high and low frailty index indicators on predicting elective surgery discharge destinations
Source: PLoS One. 2023 Apr 7;18(4):e0284206. doi: 10.1371/journal.pone.0284206 (PMC10081744; doi:10.1371/journal.pone.0284206)
Supplement: S2 Appendix — (DOCX) [file pone.0284206.s002.docx]

**S2 Appendix**

Neuralware’s^®^ Neuralworks^®^ Professional II Plus integrated neural network development environment is used for creation, training, and testing all ANN models. The backpropagation learning algorithm is used for training all of the ANNs, with a learning coefficient of 0.3 for the first hidden layer and reduced by 0.05 for each successive layer, and a momentum term of 0.4.

One hidden layer, two hidden layer, and three hidden layer architectures (only when the 2-hidden layer architecture outperformed the 1 hidden layer architecture) were evaluated for each ANN model. The specific architecture for the mFI and separate high and low mFI networks for each of the surgical specialty areas and the combination of all surgeries into a single set are given below in Table S1.

From Table S1, it may be seen that 2 hidden layer architectures were usually the best performing ANN models. Only the original mFI ANN model for the general surgery specialty performed best with a 3-hidden layer architecture, though it should be noted, that result was only 1.25% above the best 2-hidden layer architecture (34.83% accuracy versus 33.58% accuracy).

The architectures for the leave-one-out experiment in which the index value used by each ANN model was either the high impact indicator mFI or the low impact indicator mFI, all used the corresponding original mFI architecture. This is because the number of input variables was the same across all 3 ANN models and as a time saving measure. Thus, the architectures for these two ANN models for each surgical specialty are not reported separately from the original mFI architectures for each surgical specialty.

**Table S1.** ANN architectures for the different surgical specialties and mFI variable models.

| Surgical Specialty | Type of mFI used | Number of hidden layers | Nodes per layer^†^ (comma separated) |
| --- | --- | --- | --- |
| Cardiac | mFI | 2 | 6, 18, 9, 9 |
|  | high and low mFI | 2 | 7, 21, 9, 9 |
| ENT | mFI | 2 | 6, 12, 9, 9 |
|  | high and low mFI | 2 | 7, 14, 9, 9 |
| General | mFI | 3 | 6, 12, 9, 3, 9 |
|  | high and low mFI | 2 | 7, 21, 9, 9 |
| Gynecology | mFI | 2 | 6, 12, 9, 9 |
|  | high and low mFI | 2 | 7, 7, 9, 9 |
| Neurology | mFI | 2 | 6, 18, 9, 9 |
|  | high and low mFI | 2 | 7, 28, 9, 9 |
| Orthopedics | mFI | 1 | 6, 12, 9 |
|  | high and low mFI | 2 | 7, 15, 8, 9 |
| Thoracic | mFI | 2 | 6, 12, 9, 9 |
|  | high and low mFI | 2 | 7, 21, 9, 9 |
| Urology | mFI | 1 | 6, 12, 9 |
|  | high and low mFI | 2 | 7, 21, 9, 9 |
| Vascular | mFI | 2 | 6, 12, 9, 9 |
|  | high and low mFI | 1 | 7, 7, 9 |
| All (combined) | mFI | 1 | 6, 18, 9 |
|  | high and low mFI | 2 | 7, 28, 9, 9 |

^†^ Layers include the input (6 or 7) and output layer (9), with hidden layers specified in between.
